# AMPK-PPARγ-Cidec Axis Drives the Fasting-Induced Lipid Droplet Aggregation in the Liver of Obese Mice

**DOI:** 10.3389/fnut.2022.917801

**Published:** 2022-07-04

**Authors:** Hongqiang Li, Jian Sun, Bojiang Li, Aiwen Jiang, Jingli Tao, Caibo Ning, Rongyang Li, Honglin Liu

**Affiliations:** ^1^College of Animal Science and Technology, Nanjing Agricultural University, Nanjing, China; ^2^Hebei Key Laboratory of Specialty Animal Germplasm Resources Exploration and Innovation, College of Animal Science and Technology, Hebei Normal University of Science and Technology, Qinhuangdao, China; ^3^College of Animal Science and Veterinary Medicine, Shenyang Agricultural University, Shenyang, China

**Keywords:** fasting, lipid droplet, obesity, AMPK, PPARγ, Cidec

## Abstract

Intermittent fasting is one of the most common clinical treatments for the obesity, a main risk factor of the metabolic syndrome which can lead to a variety of diseases. Fasting-induced fat mobilization alters the metabolic state of lipid in the liver, predisposing to increase the hepatic lipid droplet aggregation and triglyceride levels. However, the underlying mechanisms regarding the lipid droplet aggregation in the liver after fasting remains elusive. Here, we report that a lipid droplet surface binding protein Cidec (cell death inducing DFFA like effector C) is activated by AMPK to regulate the hepatic lipid droplet fusion following fasting in obese mice. Specifically, we found that lipid droplets were significantly aggregated in the liver of high-fat-diet and *ob/ob* mice after 16 and 24 h of fasting, accompanied by the dramatically up-regulated expression of *Cidec*. Consistently, overexpression of *Cidec* in the AML12 cells resulted in the intracellular lipid droplet aggregation. Furthermore, we showed that fasting caused the up-regulated expression of AMPK, which in turn activated the transcription of *Cidec* through the transcription factor PPARγ. Altogether, our observations reveal that fasting-induced hepatic lipid droplet aggregation is mediated by the AMPK-activated expression of *Cidec* via PPARγ, extending our understanding about the molecular mechanism of the impact of fasting on the obesity and providing potential targets for the treatment of human obesity.

## Introduction

Obesity is characterized by an excessive amount of body fat that would increase the risk for many other health problems. Epidemiological studies have indicated that a high BMI (body mass index) is closely correlated with several chronic metabolism-related diseases, including NAFLD (non-alcoholic fatty liver disease), cardiovascular disease, T2D (type 2 diabetes mellitus), and different types of cancer (1, 2). Hepatic steatosis refers to the abnormal accumulation of lipids in the liver, an essential metabolic organ that governs body energy metabolism, and is the most common form of chronic liver disease (3). Patients with hepatic steatosis may have serious complications such as steatohepatitis, fibrosis, cirrhosis, liver failure, and hepatocellular carcinoma (4, 5). Although several pharmacological treatments are being developed, weight loss is still an effective treatment option for hepatic steatosis (6).

It has been reported that short-term diet restriction or fasting can prevent and reverse obesity, thus fasting has been widely used for the treatment and mechanistic studies of metabolic syndrome (7, 8). The main types of fasting interventions currently available for obesity include TRF (time-restricted feeding) and ADF (alternating day fasting). Early studies about TRF were aimed to understand the effects of food intake timing on circadian rhythms, but subsequently found that fasting has important effects on the energy metabolism (9). TRF for 4 h per day normalizes circadian rhythms, improves glucose metabolism, and reduces body weight in mice. TRF for 9 h per day reduces blood glucose levels, insulin levels, and insulin sensitivity in a type I diabetic rat model (10). In addition, ADF has been reported to have beneficial effects on the reduction of body weight, visceral fat mass, and blood lipids (11).

After fasting, the body shifts from lipid synthesis and fat storage to fat mobilization in the form of FA (fatty acid) and FA-derived ketones, resulting in the weight loss. When the cessation of food intake occurs, the body experiences several different stages of metabolic adaptation to maximize the rate of survival. Following digestion, absorption and storage of nutrients, glycogen stored in the liver is utilized to maintain the blood glucose levels. From 12 to 36 h, adipose tissue is converted from a net lipid storage organ to a lipid release organ, raising the level of non-esterified fatty acids in the blood. In the meantime, glycogen stored in the liver is almost completely emptied and gluconeogenesis becomes the main source of glucose, accompanied by the rapid degradation of muscle proteins to provide glycogenic amino acids. Excessive FFA (free fatty acid) accumulates in the liver and is re-esterified to TG (triacylglycerols), but the mechanisms by which these triglycerides are formed have not been clarified. After fasting for 48 h, ketone body production in the liver is strongly activated, partially replacing glucose as the fuel for the brain and maintaining the gluconeogenesis as well as muscle protein catabolism at low levels. Ultimately, ketone bodies turn into the primary fuel for the brain, with other tissues relying almost exclusively on fatty acids as the fuel (12). Although intermittent fasting and high-intensity interval training can be the effective approaches to reduce the body weight and fat in humans in the short to medium term (13, 14), there is still a lack of understanding of the underlying molecular mechanisms.

The CIDE protein family consists of three members, Cidea, Cideb and Cidec (also known as Fsp27, fat-specific protein of 27 kD). Analysis of the mouse CIDE proteins has shown that they play important roles in regulating lipid homeostasis including lipid storage, lipolysis and lipid secretion (15). *Cidea* knockout mice exhibit enhanced lipolysis, reduced serum triglycerides and free fatty acids, similar to the results of *Cidea* knockdown in human white adipose tissue. Cidec is highly expressed in white adipose tissue, and *Cidec* knockout mice display less fat storage, increased lipolysis, and reduced lipid droplet volume (16). Cideb promotes lipid accumulation during normal diet, whereas Cidea and Cidec cause hepatic fat accumulation in fasted conditions. These findings suggest that CIDE family proteins take critical part in regulating lipid metabolism in adipose and liver tissues.

In the present study, we applied high-fat-diet obese mouse model to explore the mechanism of fasting-induced lipid metabolism in the liver. We observed that fasting promoted the accumulation of lipid droplets in the liver of obese mice, along with the up-regulated expression of *Cidec*. We further found that fasting activated the expression of AMPK, followed by regulating the transcription of *Cidec* through the transcription factor PPARγ. These findings uncovered a molecular network of hepatic lipid aggregation caused by fasting in the obese mice.

## Materials and Methods

### Animals

Four-week-old male C57BL/6J mice were purchased from the Medical Laboratory Animal Center of Nanjing Medical University and 4-week-old male ob/ob mice were purchased from the Institute of Model Animal Research, Nanjing University. The mice were housed at a population density of five per cage on a 12/12 h light/dark cycle with constant temperature (24 ± 2°C) and humidity (40–60%), free access to food and water. All experimental protocols involving mice were approved by the Animal Care and Use Committee of Nanjing Agricultural University, and all experiments were conducted in compliance with the guidelines of the local animal ethical committee and the Animal Care and Use Committee of Nanjing Agricultural University.

### Establishment of Obese Mouse Model

The mice were grouped by feeding with normal chow (control) and high-fat chow for 8 weeks, respectively. The formula of normal diet (ND) consists of 9% wheat, 10% bran, 8% soybean meal, 35% corn, 8–12% fish meal (species fish), 3% egg, 2% yeast, 2% soybean oil, 15% malt root, 1% soybean meal and 4% premix. High-fat diet (HFD) formula consists of 12% lard, 2% cholesterol, 0.2% propyl, 0.5% bile salt, and 85.3% regular feed.

### Experimental Procedure for Fasting

The ND and HFD mice were divided into five groups, respectively, with 10 in each group. Fasting was performed for 0 h (6 p.m.), 8 h (6 p.m. to 2 a.m.), 16 h (6 p.m. to 10 a.m. of the next day), 24 h (6 p.m. to 6 p.m. of the next day), 32 h (6 p.m. to 2 a.m. of the next day), with 0 h of fasting as a control. During the fasting treatment, each group of mice had free access to water.

### RNA Isolation and Real-Time PCR

Total RNA was extracted from mouse liver and cell samples using Trizol lysate (Thermo Fisher Scientific), and then reversed to cDNA using Hifair II 1st Strand cDNA Synthesis Kit (Shanghai Yeasen Biotech). The real-time PCR system was 20 μl in total which included 10 μl SYBR PCR mix, 2 μl cDNA template, 0.4 μl sense primers and anti-sense primers (The primer information is presented in Table 1) and 6.8 μl deionized water. PCR reaction programme was set as follows: pre-denaturation at 95°C for 5 min, followed by 40 cycles of 95°C for 10 s, 60°C for 30 s; collection of melting curves at 95°C for 15 s, 60°C for 60 s, and 95°C for 15 s. β-actin was used as an internal reference to correct the relative expression of genes, and 2^−Δ*ΔCt*^ method was used for statistical analysis of gene expression level. GraphPad Prism 5 software was used to analyze the level of data significance among different groups.

**Table 1:** Primer list for fluorescent quantitative PCR.

**Gene names**	**Primer sequence (5^**′**^-3^**′**^)**	**Annealing Tm (**°**C)**
*Cpt1a*	F:TGAGTGGCGTCCTCTTTGG R:CAGCGAGTAGCGCATAGTCATG	59.0
*Srebp1c*	F:GGAGCCATGGATTGCACATT R:GGCCCGGGAAGTCACTGT	60.0
*Acc*	F:TGACAGACTGATCGCAGAGAAAG R:TGGAGAGCCCCACACACA	59.0
*Scd1*	F:CCGGAGACCCCTTAGATCGA R:TAGCCTGTAAAAGATTTCTGCAAACC	61.4
*Atgl*	F:CTTCCTCGGGGTCTACCACA R:GCCTCCTTGGACACCTCAATAA	59.7
*Hsl*	F:TTCTCCAAAGCACCTAGCCAA R:TGTGGAAAACTAAGGGCTTGTTG	58.7
*Cidec*	F:ACTTGTGCCGTCTTCCGTG R:GCTCGCTTGGTTGTCTTGATT	59.7
*PPARγ*	F:TTCAAGGGTGCCAGTTTCG R:GGGAGGCCAGCATCGTG	60.0
*β-actin*	F:TGAACCCTAAGGCCAACCG R:GGAGAGCATAGCCCTCGTAGAT	59.5

### Preparation of Tissue Sections

The tissue samples were fixed in 4% paraformaldehyde and wrapped with an embedding machine and cut to a thickness of 3 μl with a microtome. The sections were then placed in 0.5% hydrochloric acid alcohol for 2 s, rinsed by clean water for 20 s, and incubated in hot water for 30 s. After that, the sections were put into eosin staining solution for 2 s, transferred to a glass jar to rinse by clean water for 10 s, followed by dehydration using a gradient concentration of ethanol for 2 min. Finally, the sections were placed in xylene for 2 min prior to sealing with neutral gum.

### Western Blot

The protein was extracted from tissues or cells using a protein extraction kit (Shanghai Biyuntian Biotech), and the protein concentration was determined by BCA protein assay kit (Shanghai Biyuntian Biotech). 15–20 μg of total protein was mixed with 5 × SDS loading buffer and protease inhibitor, and then denatured by boiling at 100°C for 5 min. Proteins were separated by pre-electrophoresis at a constant voltage of 60 V for 20 min, followed by electrophoresis at a constant voltage of 100 V for 40–90 min according to the size of the target proteins. Separation gel was then transferred to the PVDF membrane at a constant voltage of 100 V for 90 min. The blots was washed three times with TBST, blocked with solution containing 5% BSA at room temperature for 1 h, and incubated with primary antibodies (AMPK antibody, phosphorylated AMPK antibody, PPARγ antibody, Cidec antibody, GAPDH antibody and α-tubulin antibody were purchased from Cell Signaling Technology) at 4°C overnight. After three times of wash in TBST, the blots were incubated with appropriate HRP-conjugated secondary antibodies. The chemiluminescence signals were developed using an ECL chromogenic solution (Advansta) and acquired by LAS-4000 gel imaging system.

### Cell Culture

Cells were cultured in 10% FBS complete DMEM medium (GIBCO, Grand Island, NY, USA) with an additional 100,000 units/L penicillin sodium, and 100 mg/l streptomycin sulfate (Hyclone) in a 37°C, 5% CO_2_ incubator. The medium was aspirated when the cells reached 70–90% density. AICAR (selleck)-treated and Dorsomorphin (selleck)-treated AML12 cells were collected after 24 h of treatment.

### Luciferase Reporter Assay

The pGL3-Cidec, pGL3-Basic, and pGL3-Control plasmids were diluted to 200 ng/μl, and the internal reference plasmid (TK) was diluted to 20 ng/μl. Then 1,000 ng of pGL3-Cidec and 50 ng of internal reference plasmid were co-transfected into cells, with pGL3-Basic and pGL3-Control plasmids as negative and positive controls, respectively. At 48 h after transfection, cells were harvested to measure the luciferase activity.

### Oil Red O Staining

Cells were seeded in a slide-coated 24-well plate after 24 h of transfection, and then stained with 0.5% oil red O (Sigma-Aldrich) for 10 min. Afterwards, cells were washed by PBS and stained with 5 μM DAPI (Sigma-Aldrich) for 10 min, followed by imaging with a fluorescence microscope (Zeiss Axio Scope A1). The micro digital measurement and analysis system was used to count the number of fat droplets in 30 randomly selected cells, and measure the diameter of fat droplets in 70 randomly selected fat droplets.

### Statistical Analysis

Data were expressed as means ± SD, unless otherwise stated. All analyses were performed using GraphPad Prism (Version 5.0). Statistical comparisons were made with two-tailed Student's t test. Changes were considered statistically significant when *P* < 0.05.

## Results

### Fasting Changes the Amount and Morphology of Lipid Droplets in the Liver of Obese Mice

**Figure 1:**
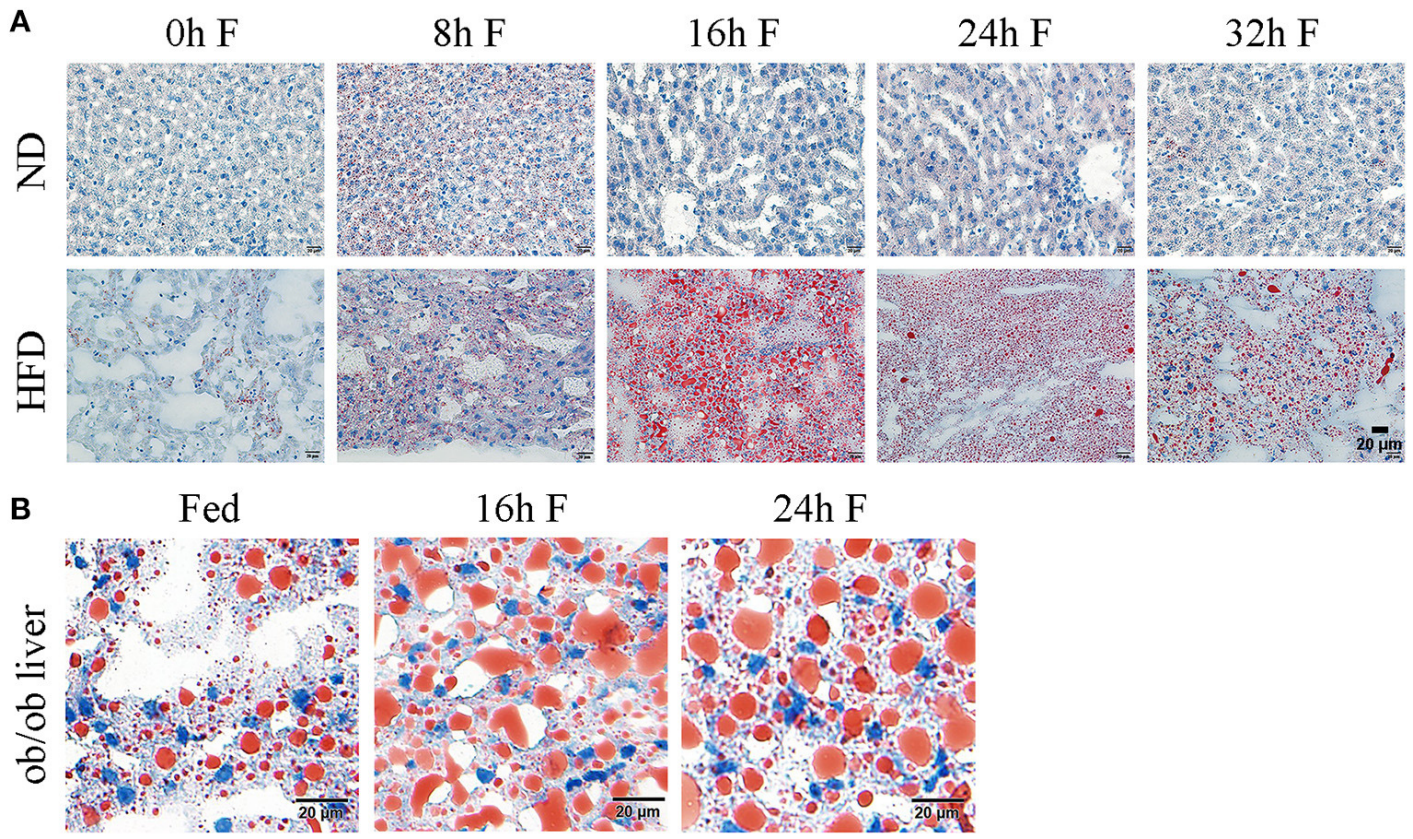
Effects of fasting on the lipid droplet dynamics in the liver of obese mice. **(A)** Representative images of hepatic lipid droplet in HFD mice. ND, normal diet; HFD, high fat diet. Liver sections were stained with oil red O to display the lipid droplets (red) and counterstained with hematoxylin to display the nuclei (blue). Scale bar, 20 μm. **(B)** Representative images of hepatic lipid droplet in ob/ob mice. Liver sections were stained with oil red O to display the lipid droplets (red) and counterstained with hematoxylin to display the nuclei (blue). Scale bar, 20 μm.

After 8 weeks of feeding, the average body weight of mice in ND (normal diet) group was 20.79 ± 1.31 g, while it reached 34.83 ± 3.26 g in HFD group, reflecting a 67.53% increase in the body weight (data not shown). According to the reported criteria that more than 20% average weight gain could be considered as the diet-induced obesity, the obese mouse model in our study was successfully established. To investigate the impact of fasting on the hepatic lipid metabolism, the histological sections of livers from ND and HFD mice were stained with oil red O to observe the changes of lipid droplets. As shown in Figure 1, the morphology and number of lipid droplets in the liver of ND mice did not apparently alter at different time points after fasting, except that a number of small lipid droplets were present at 8 h of fasting. By contrast, the amount of lipid droplets in the livers of HFD mice was significantly increased after 8 and 16 h of fasting, with the presence of unusually large and small lipid droplets, and the large lipid droplets displayed a light staining. At 24 h of fasting, the number of lipid droplets remained constant, but the large lipid droplets became smaller with deeper staining compared to 16 h. Until 32 h of fasting, the number of lipid droplets began to decrease (Figure 1A). Notably, the number and volume of lipid droplets were substantially increased in HFD mice at each time point of fasting compared to those in ND mice (Figure 1A). To further verify whether lipid droplet fusion induced by fasting was a general phenotype in obese mice, we also observed the lipid droplet morphologies at 16 and 24 h of fasting in *ob/ob* mice. The oil red O staining of liver sections revealed that the lipid droplets were apparently increased with light staining in *ob/ob* mice at 16 and 24 h of fasting (Figure 1B), indicating that fasting promoted lipid droplet fusion in both diet- and genetically-induced obesity mouse models. Collectively, these data indicate that fasting has a general effect on the hepatic lipid deposition in obese mice.

### Cidec Expression Is Induced by Fasting in HFD Mice

**Figure 2:**
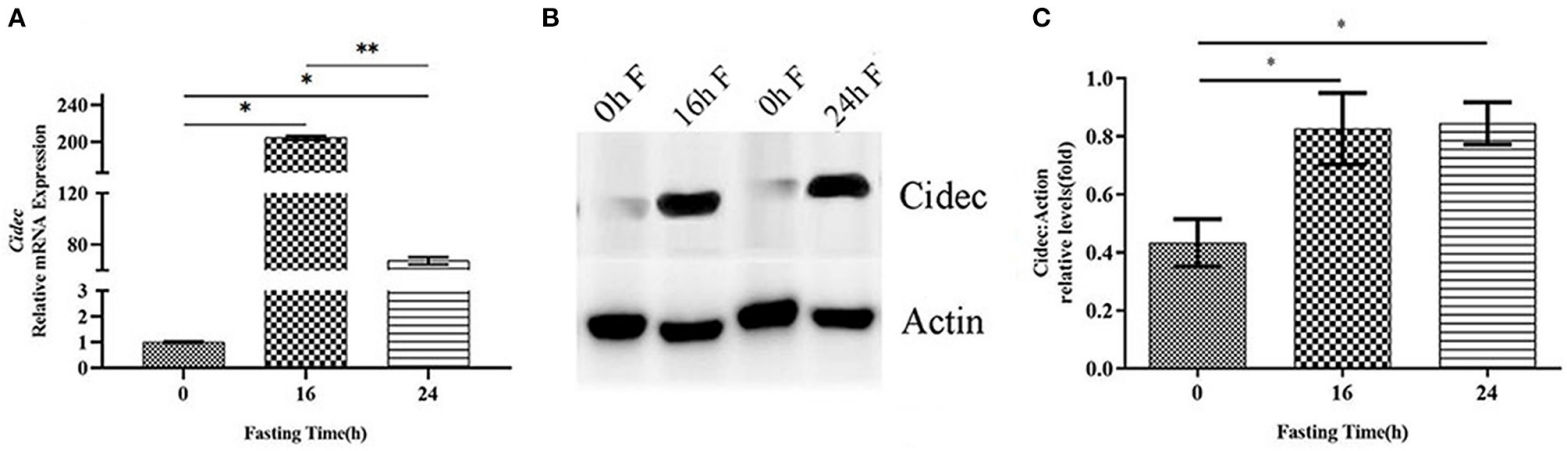
Effects of fasting on the Cidec expression in HFD mice. **(A)** mRNA expression levels of Cidec in the liver of HFD mice subjected to 16 h and 24 h fasting. **(B)** Protein expression levels of Cidec in the liver of HFD mice subjected to 16 h and 24 h fasting. **(C)** Histogram plotted with the corresponding grayscale values in the **(B)**. Data in **(A,C)** were analyzed by t-test, with actin as the internal control. *P < 0.05, **P < 0.01.

Previous studies have shown that Cidec takes a critical part in promoting lipid droplet fusion and development (17), we thus examined the involvement of Cidec in the lipid droplet dynamics in HFD mice under fasting condition. We detected the mRNA and protein expression levels of Cidec in HFD mice at different time points of fasting. As shown in Figure 2A, mRNA expression of *Cidec* was considerably upregulated at both 16 h (*P* < 0.05) and at 24 h (*P* < 0.05) after fasting. Consistently, significant increases of Cidec protein levels were observed at 16 and 24 h of fasting compared to 0 h (*P* < 0.05; Figures 2B,C), suggesting that fasting in the context of obesity promotes the expression of *Cidec*.

### Overexpression of Cidec Leads to the Fusion of Lipid Droplets in AML12 Cells

**Figure 3:**
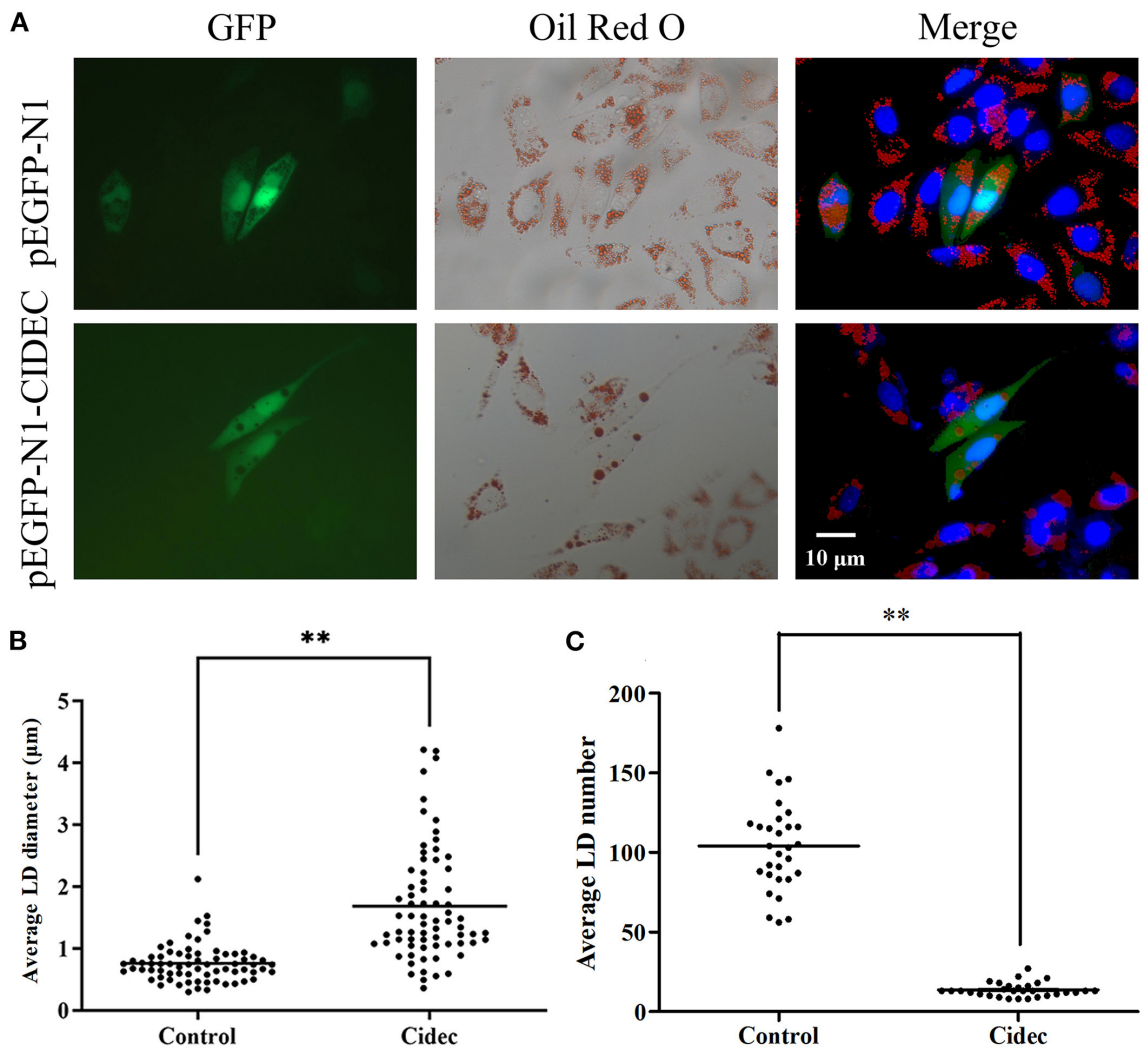
Effects of Cidec overexpression on the lipid droplet morphology in AML12 cells. **(A)** Representative images of lipid droplets in AML12 cells transfected with pIRES2-EGFP and pIRES2-EGFP-Cidec. Cells were stained with oil red O and counterstained with DAPI. GFP signal (green), oil red O (red), DAPI (blue). Scale bar, 10 μm. **(B)** The diameter of lipid droplets were measured in AML12 cells transfected with pIRES2-EGFP and pIRES2-EGFP-Cidec. **(C)** The nunmber of lipid droplets were quantified in AML12 cells transfected with pIRES2-EGFP and pIRES2-EGFP-Cidec. Data in **(B,C)** were analyzed by t-test. **P < 0.01.

Given that we have observed the correlation between expression of *Cidec* and lipid droplet change in the liver, we then tested if overexpression of *Cidec* could directly affect the dynamics of lipid droplets in AML12 cells. As shown in Figure 3A, the controls cells transfected with pIRES2-EGFP vector exhibited more number but less volume of lipid droplets than those transfected with pIRES2-EGFP-Cidec. Quantitatively, the diameter of lipid droplets in Cidec-overexpressed cells was markedly longer than that in controls (*P* < 0.01; Figure 3B). While the average number of lipid droplets was reduced in Cidec-overexpressed cells compared to the controls (*P* < 0.01; Figure 3C), revealing that Cidec can promote the lipid droplet fusion.

### Fasting Activates AMPK Expression in HFD Mice

**Figure 4:**
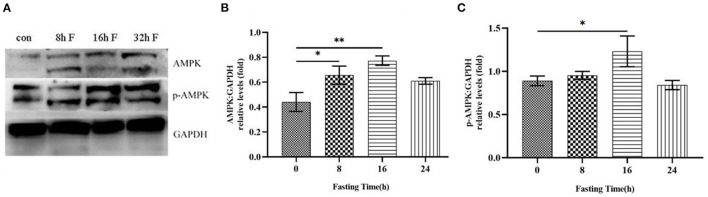
Effects of fasting on the total protein level and phosphorylation level of AMPK in HFD mice. **(A)** Western blot analysis was performed to show the changes of AMPK protein level and phosphorylation level in HFD mice after different time of fasting. **(B)** Histogram plotted by the grayscale values of AMPK relative to Gapdh. **(C)** Histogram plotted by the grayscale values of p-AMPK relative to Gapdh. *P < 0.05, **P < 0.01.

Adenosine monophosphate-activated protein kinase (AMPK) is a cellular energy sensor that is activated at low energy states to restore the energy, depending on the ratios of AMP/ATP and NAD^+^/NADH. After fasting, the starvation increases the AMP/ATP ratio *in vivo*, we thus hypothesized that AMPK might be activated under this condition. To confirm this possibility, the liver tissues from HFD mice at different fasting time points were collected to detect the total protein levels and phosphorylation levels of AMPK by immunoblotting analysis. The results showed that total protein levels of AMPK were gradually up-regulated with the prolonged fasting time (Figures 4A,B). Similarly, the phosphorylation levels of AMPK were also increased over time, with the highest level at 16 h of fasting (Figures 4A,C).

### Effects of AMPK Activity on the Expression of Lipid-Associated Genes in AML12 Cells

**Figure 5:**
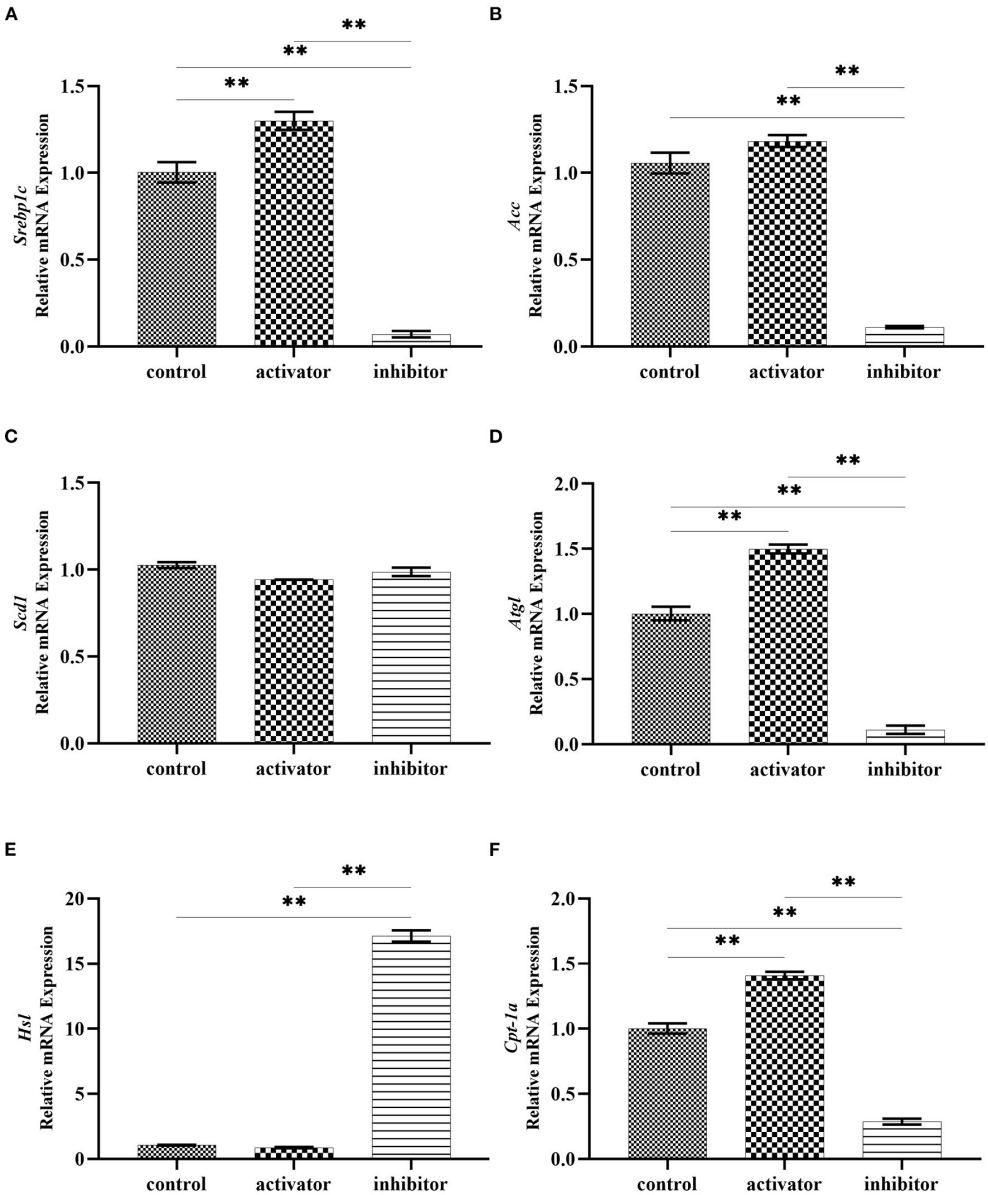
Effects of AMPK activity on the expression of lipid synthesis and decomposition related genes. **(A–C)** mRNA expression of genes related to lipid synthesis in Acadesine-treated and Dorsomorphin-treated AML12 cells. **(D–F)** mRNA expression of genes related to lipid decomposition in Acadesine-treated and Dorsomorphin-treated AML12 cells. β-actin was used as the internal control. Data were analyzed by t-test. **P < 0.01.

To determine the effect of AMPK on the lipid deposition and droplet fusion, we treated AML12 cells with AMPK activator and inhibitor to detect the expression of genes related to lipid synthesis and lipolysis. Quantitative RT-PCR results revealed that the expression of lipid synthesis genes did not significantly change after AMPK activation by Acadesine treatment, but exhibited a decreased trend after AMPK inhibition by Dorsomorphin (compound C) treatment (Figures 5A–C). Among them, *Srebp1c* (sterol regulatory element binding protein 1C) and *Acc* (acetyl CoA carboxylase) displayed a substantial down-regulated expression in AMPK-inhibited cells compared to the controls (Figures 5A–C), indicating that the activation of AMPK had no effect on the lipid synthesis. In addition, as shown in Figures 5D–F, TG catabolism key gene *Atgl* (adipose triglyceride lipase) and FA oxidation key gene *Cpt1a* (carnitine palmitoyltransferase 1A) were elevated, but the lipid catabolism related gene *Hsl* was decreased in the presence of AMPK activator Acadesine. Conversely, the expression patterns of these genes were reversed when inhibition of AMPK activity, indicating that lipid catabolism is enhanced by the activation of AMPK.

### Fasting Promotes PPARγ Expression in HFD Mice

**Figure 6:**
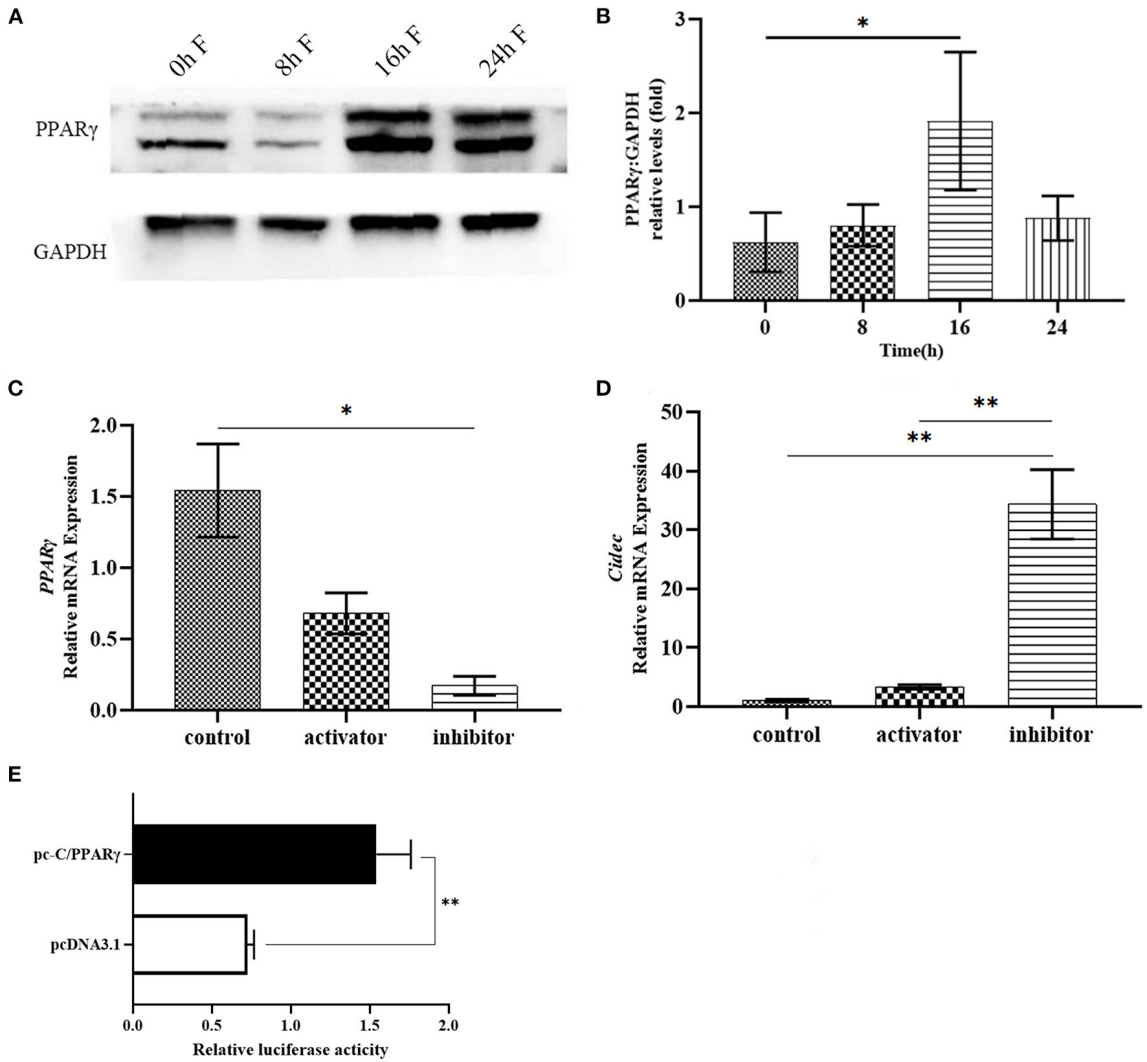
Effects of fasting on the expression of PPARγ in the liver of HFD mice. **(A)** Protein levels of PPARγ in the liver of HFD mice after fasting at different times. **(B)** Histogram plotted by the grayscale values of PPARγ relative to Gapdh. **(C,D)** Activation and inhibition of AMPK on the mRNA expression of PPARγ and Cidec. β-actin was used as the internal control. **(E)** Binding of PPARγ to the promoter region of Cidec was assessed by luciferase activity. Data were analyzed by t-test. *P < 0.05, **P < 0.01.

As bioinformatics analysis predicted that PPARγ is a potential transcription factor for *Cidec* expression, we proposed that PPARγ might regulate the transcription of *Cidec* under the fasting condition, leading to the lipid droplet fusion and volume change. To this end, we examined the protein expression of PPARγ following fasting in the liver of HFD mice. As shown in Figures 6A,B, with the progression of fasting time, the PPARγ protein amount showed an increased trend, with the highest level at 16 and 24 h of fasting, which confirmed the enhanced expression of PPARγ induced by fasting. Then, AML12 cells were treated with AMPK activator and inhibitor to detect the transcript levels of *PPAR*γ and *Cidec*. We observed that *PPAR*γ expression was decreased but *Cidec* expression was increased after treatment with both AMPK activator and inhibitor (*P* < 0.05; Figures 6C,D), suggesting that AMPK is the upstream regulator of *Cidec* and PPARγ in AML12 cells.

To further verify the potential effect of PPARγ on the transcriptional regulation of *Cidec*, plasmids containing the sequences of full-length *PPAR*γ and *Cidec* promoter region were co-transfected into 293T cells to test their interaction using dual luciferase reporter system. The result showed that the luciferase activity of co-transfected cells was considerably higher than that of the controls (*P* < 0.01; Figure 6E), indicating that PPARγ can bind to the promoter region of *Cidec* and thus regulate its transcriptional expression.

### Starvation Triggers the Activation of AMPK-PPARγ-Cidec Axis in AML12 Cells

**Figure 7:**
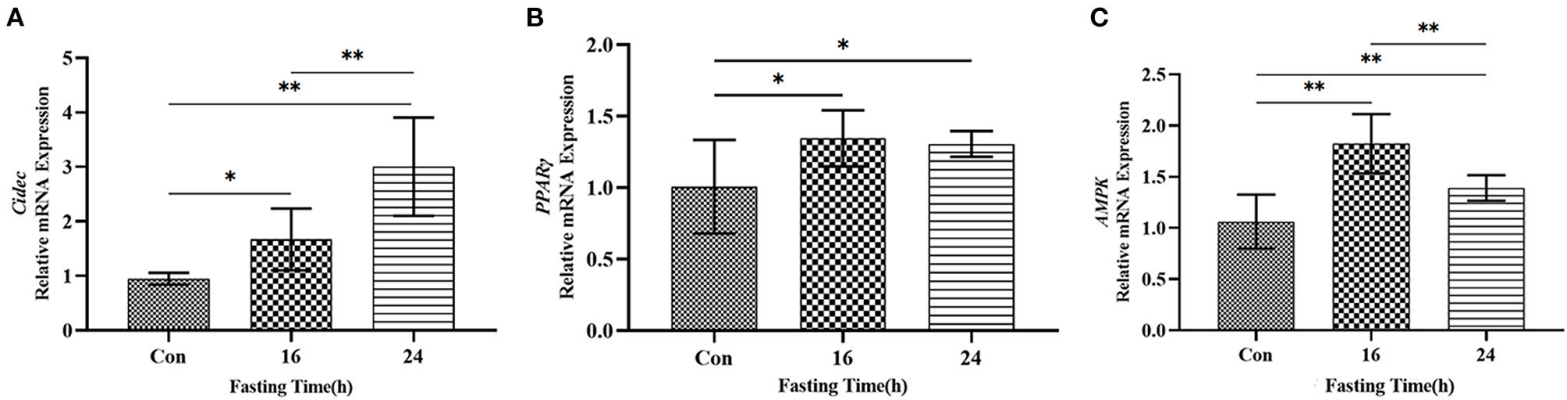
Expression of Cidec, PPARγ and AMPK in a starvation cell model. **(A)** mRNA expression of Cidec in starved AML12 cells. **(B)** mRNA expression of PPARγ in starved AML12 cells. **(C)** mRNA expression of AMPK in starved AML12 cells. T-test analysis was used for data statistics. β-actin was used as the internal control. Data were analyzed by t-test. *P < 0.05, **P < 0.01.

Lastly, we tested the expression of *Cidec, AMPK* and *PPAR*γ in AML12 cells cultured in sugar-free medium which can induce the starvation status in cells. As shown in Figure 7, mRNA levels of *Cidec, AMPK* and *PPAR*γ in AML12 cells as assessed by qRT-PCR were all upregulated after 16 h and 24 h of *in vitro* culture in starved media (*P* < 0.05, *P* < 0.01; Figures 7A–C), which indicates that AMPK-PPARγ-Cidec axis is also activated by starvation at cellular level.

## Discussion

During fasting, liver enters a state of physiological steatosis and lipids are stored in the lipid droplet for energy provision to exert vital cellular functions (18, 19). At this time, hepatic triglyceride is synthesized by the non-esterified fatty acids from the hydrolysis of triglyceride stored in adipose tissue, dietary fatty acids from intestinal celiac residues, and newly synthesized fatty acids through lipogenesis (19). Proteomic analysis of hepatic lipid droplets under different nutritional conditions demonstrates that the abundance of proteins in the lipid droplets varies dramatically between fasting and refeeding mice, with altered metabolic properties of the liver. In addition, the morphological properties of lipid droplets are dynamically regulated by the metabolic state of the liver, and lipid droplets may take a part in coordinating various metabolic activities within hepatocytes (20). In line with these previous studies, we found that lipid droplet was accumulated in the liver of obese mice after fasting, accompanied by the elevated expression of *Cidec*.

Cidec has been found to regulate the fusion of small multi-compartmental lipid droplets to large uni-compartmental ones (21). On the contrary, adipocyte-specific knockdown of Cidec leads to the increased lipolysis (22, 23). Concordantly, knockout of Cidec in mice increases the number of lipid droplets in white adipocytes, and thus protects mice from diet-induced obesity and insulin resistance. During lipid droplet fusion, Cidec interacts with Plin1 through the CIDE-N terminus at the nascent lipid droplet contact site to promote lipid transfer and single-compartment lipid droplet formation (24). Also, the CIDE-C terminus is essential during lipid droplet growth (25). In adipocytes, Cidec regulates the catalytic capacity of lipid-related enzymes, which in turn promotes lipid droplet morphological changes and lipolysis (26). For instance, Cidec prevents lipolysis by interacting with Atgl, a rate-limiting enzyme that modulates the lipolysis process (27). Consistent with the regulatory role of Cidec in lipid droplet growth, Cidec knockout mice are leaner and are protected from diet-induced obesity and insulin resistance. Whereas overexpression of Cidec promotes triglyceride accumulation in adipocytes and hepatocytes (28). These studies demonstrate that Cidec is a key mediator of lipid droplet dynamics and that reduced expression of Cidec accelerates lipid utilization. Notably, in our study, the role of *Cidec* in the lipid droplet fusion was verified in the AML12 cells overexpressing *Cidec* and AML12 cells induced to starvation status, which suggests that both *in vivo* fasting and *in vitro* starvation result in the increased expression of *Cidec*.

Cidec expression is controlled by multiple signaling pathways, which are interconnected to form a transcriptional regulatory network. It has been found that Cidec transcription is up-regulated mainly through the cAMP/PKA-CREB-CRTC2 (CREB-regulated transcription coactivator 2) signaling pathway to participate in the lipolysis during the initial phase of fasting in mice, and is down-regulated to produce energy by increased fatty acid supply during the late period of fasting (29–31). However, our findings showed that PPARγ expression is up-regulated in the liver of mice under fasting condition and starved AML12 cells, revealing the relationship between the expression of PPARγ and Cidec, which is consistent with previous studies that growth hormone regulates lipolysis by activating MEK/ERK and STAT5-dependent signaling pathways to inhibit Cidec expression, in coordination with PPARγ transcriptional activity, as well as Cidec is transcriptionally controlled by PPARγ during adipocyte differentiation.

AMPK is a serine/threonine protein kinase complex that is widely expressed in almost all eukaryotes (32, 33). Activated AMPK has a dual function on cellular metabolism when cells sensitize the stress and reduced ATP levels. AMPK activation inhibits anabolic pathways to reduce ATP consumption, including the synthesis of lipid, sterol, glycine, RNA, protein, as well as the cell cycle, and promotes catabolic pathways to replenish ATP, including autophagy, glucose uptake and utilization, mitochondrial biosynthesis, and lipid utilization (34). AMPK regulates gene transcription through several different pathways in response to glucose starvation and oxidative stress. In mouse embryonic fibroblasts, glucose starvation activates AMPK, which then induces the expression of antioxidant genes such as catalase, Sod2 and Ucp2 *via* PPARγ and PGC-1α (35). AMPK is also required for upregulation of PGC-1α *via* p38 in cancer cells, thus promoting mitochondrial biogenesis (36). In addition, increased ROS induced by glucose starvation further activates the LKB1-AMPK signaling pathway, hence promoting autophagic degradation of Keap1 (keloid epichlorohydrin-associated protein 1) (37). In our study, we discovered that AMPK and its phosphorylation levels were significantly upregulated in the liver of HFD mice after fasting and starved AML12 cells, in agreement with the notion that energy deficiency can induce an increase in AMPK expression both *in vivo* and *in vitro*. Insulin resistance has been reported to cause the increased expression of Cidec and promote the translocation of Cidec into the nucleus to interact with AMPKα2 (38). Also, upregulation of Cidec in adipocytes is accompanied by a decrease in AMPKα level, which promotes the differentiation of adipocytes (39).

To sum up, our findings provide several lines of evidence demonstrating that fasting drives the lipid droplet accumulation in obese mice, which is mediated by the AMPK-PPARγ-Cidec signaling axis, contributing to understand the molecular basis of the fasting effect on the obesity and develop the feasible strategies to prevent and treat the obesity in humans.

## Data Availability Statement

The original contributions presented in the study are included in the article/supplementary material, further inquiries can be directed to the corresponding author/s.

## Ethics Statement

The animal study was reviewed and approved by the Animal Care and Use Committee of Nanjing Agricultural University.

## Author Contributions

HLiu conceived the research. HLi, JS, BL, AJ, JT, CN, and RL conducted the experiments. HLi and HLiu analyzed the data and wrote the manuscript. All authors contributed to the article and approved the submitted version.

## Funding

This work was supported by the National Natural Science Foundation of China (31702101) and Hebei Province Talent Training Project (A201901055).

## Conflict of Interest

The authors declare that the research was conducted in the absence of any commercial or financial relationships that could be construed as a potential conflict of interest.

## Publisher's Note

All claims expressed in this article are solely those of the authors and do not necessarily represent those of their affiliated organizations, or those of the publisher, the editors and the reviewers. Any product that may be evaluated in this article, or claim that may be made by its manufacturer, is not guaranteed or endorsed by the publisher.
